# Silver Nanoparticle-Based Combinations with Antimicrobial Agents against Antimicrobial-Resistant Clinical Isolates

**DOI:** 10.3390/antibiotics11091219

**Published:** 2022-09-08

**Authors:** Areej M. Alotaibi, Nasser B. Alsaleh, Alanoud T. Aljasham, Essam A. Tawfik, Mohammed M. Almutairi, Mohammed A. Assiri, Musaed Alkholief, Mashal M. Almutairi

**Affiliations:** 1 Department of Pharmacology and Toxicology, College of Pharmacy, King Saud University, Riyadh 11451, Saudi Arabia; 2 National Center of Biotechnology, Life Science and Environment Research Institute, King Abdulaziz City for Science and Technology (KACST), Riyadh 12354, Saudi Arabia; 3 Department of Pharmaceutics, College of Pharmacy, King Saud University, Riyadh 11495, Saudi Arabia

**Keywords:** antimicrobial resistance, engineered nanomaterials, metal nanoparticles, cytotoxicity, antimicrobial agents, synergism

## Abstract

The increasing prevalence of antimicrobial-resistant (AMR) bacteria along with the limited development of antimicrobials warrant investigating novel antimicrobial modalities. Emerging inorganic engineered nanomaterials (ENMs), most notably silver nanoparticles (AgNPs), have demonstrated superior antimicrobial properties. However, AgNPs, particularly those of small size, could exert overt toxicity to mammalian cells. This study investigated whether combining AgNPs and conventional antimicrobials would produce a synergistic response and determined the optimal and safe minimum inhibitory concentration (MIC) range against several wild-type Gram-positive and -negative strains and three different clinical isolates of AMR *Klebsiella pneumoniae*. Furthermore, the cytotoxicity of the synergistic combinations was assessed in a human hepatocyte model. The results showed that the AgNPs (15–25 nm) were effective against Gram-negative bacteria (MIC of 16–128 µg/mL) but not Gram-positive strains (MIC of 256 µg/mL). Both wild-type and AMR *K. pneumoniae* had similar MIC values following exposure to AgNPs. Importantly, co-exposure to combinations of AgNPs and antimicrobial agents, including kanamycin, colistin, rifampicin, and vancomycin, displayed synergy against both wild-type and AMR *K. pneumoniae* isolates (except for vancomycin against AMR strain I). Notably, the tested combinations demonstrated no to minimal toxicity against hepatocytes. Altogether, this study indicates the potential of combining AgNPs with conventional antimicrobials to overcome AMR bacteria.

## 1. Introduction

The high prevalence of antimicrobial-resistant (AMR) bacterial strains represents a major challenge to healthcare systems leading to increased morbidity and mortality rates globally [1,2,3,4,5]. Antimicrobial resistance is a microorganism’s ability to grow and survive even in the presence of an antimicrobial agent at a concentration that is typically adequate to inhibit their growth or eradicate them [6]. Currently, over 70% of bacterial infections are resistant to conventional antimicrobial agents which are considered the “gold standard” for the management of relevant diseases [7,8]. Unfortunately, the rapid evolution of AMR bacteria has overtaken the development rates of new antimicrobials, and in some cases, resistance may develop soon after their clinical approval [5,9,10]. In 2017, the World Health Organization (WHO) issued the first list of antimicrobial agent-resistant microorganisms posing a threat to human health, emphasizing the urgent need for unraveling novel antimicrobials and therapeutic modalities. This list includes extended spectrum β-lactamase (ESBL)-producing *Enterobacteriaceae* as a priority 1 (critical) pathogen [11]. In Saudi Arabia, several studies have pointed out the risk of increasing prevalence of AMR pathogens in local hospitals [12,13,14]. Remarkably, a recent study demonstrated the high prevalence of infection caused by these AMR bacteria, particularly *Klebsiella pneumoniae* (*K. pneumoniae*), which was associated with high mortality [15]. Another study found that the second most frequently isolated AMR bacteria from Saudi hospitals were *K. pneumoniae*, with a significant percentage being ESBL-positive [16]. As a result, clinicians have had to consider treatment regimens based on combinations of medications with inadequate activity or to reuse some old and abandoned antimicrobials, such as colistin, that are known to have several adverse reactions [17,18,19,20]. As a result, the development of novel antimicrobial agents is critically warranted to fight these microorganisms with minimal and tolerated toxicity.

Advances in nanotechnology and nanomaterials have opened new horizons for innovation in a broad range of fields and industry sectors, including energy, electronics, communication, transport, agriculture, food, biotechnology, and healthcare [21]. Engineered nanomaterials (ENMs) are precisely synthesized materials with at least one dimension between 1 and 100 nanometers (nm) [22]. Importantly, emerging evidence has demonstrated the superior antimicrobial properties of metal and metal oxide ENMs, including silver, gold, platinum, and zinc oxide nanoparticles (NPs), against a wide range of pathogens [23]. Indeed, it has been shown that exposure to these ENMs may help overcome AMR bacteria and may thus revolutionize therapy [24]. This advantage is driven largely by the ultra-small size of such ENMs and their large surface-to-volume ratio, which could enable direct interaction with the bacteria’s molecular machineries [24,25]. Notably, the use of silver NPs (AgNPs) has been broadly expanded over the past few years due to their superior antimicrobial properties against a wide range of Gram-positive and -negative bacterial strains compared to other ENMs, in addition to being cost-effective [25,26,27]. It has previously been shown that AgNPs mediate their antimicrobial activity via multiple molecular mechanisms spontaneously, including generation of reactive oxygen species (ROS), direct interaction with and rupture of biological membranes, and internalization of NPs and release of silver ions (Ag^+^), thereby acting through a Trojan horse mechanism [26,27,28,29]. Importantly, previous studies have demonstrated that AgNPs may eliminate AMR bacteria and overcome resistance mechanisms, such as inhibition of biofilm formation and modulating the microbial influx/efflux pumps that might develop by bacteria against antimicrobials [30,31,32,33].

The same advantage of using ENMs to substantiate toxicity against AMR bacteria through their large surface-to-volume ratio may pose toxicity to mammalian cells, and indeed, this represents one of the grand challenges in the field of nanomedicine [34]. To overcome the cytotoxicity of metal ENMs, some studies have demonstrated that combining metal ENMs with conventional antimicrobials may help minimize the toxicity of both agents toward mammalian cells through reducing the requirement for high-dosage regimens while enhancing the bactericidal outcomes. Hence, combining these two treatment modalities represents a promising approach, particularly against AMR bacterial strains [9,29]. There have been previous studies investigating AgNP-based combination therapy with different antimicrobial agents against a wide spectrum of bacteria including AMR ones; however, a discrepancy exists between the reports with regard to the used strains, physicochemical properties of AgNPs, sensitivity to antimicrobial agents, assessment of toxicity in mammalian cells, etc. [35,36,37,38,39]. Therefore, there is a need for a comprehensive study that evaluates AgNP-based combinations with different classes of conventional antimicrobial agents against a wide range of standard and AMR bacteria including the cytotoxicity of the relevant combinations to mammalian cells.

Recently, some studies in Saudi Arabia indicated that AgNPs might strengthen or restore the antimicrobial efficacy of conventional antimicrobial agents, either additively or synergistically, against clinical isolates of resistant bacterial strains such as methicillin-resistant *Staphylococcus aureus* (MRSA)*,* vancomycin-resistant *Enterococci* (VRE), *Escherichia coli* (*E. coli*), and *Pseudomonas aeruginosa* (*P. aeruginosa*) [37,40,41]. However, to the best of our knowledge, there have been no reports to date on the potential use of AgNP-based combinations with different classes of conventional antimicrobial agents as novel antimicrobial candidates in the treatment of AMR ESBL-positive *K. pneumoniae* local isolates. In this study, it was hypothesized that using a combination therapy of AgNPs and conventional antimicrobial agents of different classes (and hence different mechanisms of antimicrobial action), including ampicillin, kanamycin, vancomycin, ciprofloxacin, colistin, and rifampicin, would produce a synergistic effect with tolerated toxicity to mammalian cells. Accordingly, the antimicrobial activity of AgNPs alone and in combination with conventional antimicrobials was evaluated against wild-type Gram-positive and -negative bacteria as well as AMR ESBL-positive *K. pneumoniae* isolated from a local tertiary hospital in Saudi Arabia. In addition, the cytotoxic effect of the AgNP-based antimicrobial combinations against a human liver cell model was evaluated to gain some insight into the safety of the tested combinations.

## 2. Results and Discussion

### 2.1. Characterization of AgNPs

**Figure 1:**
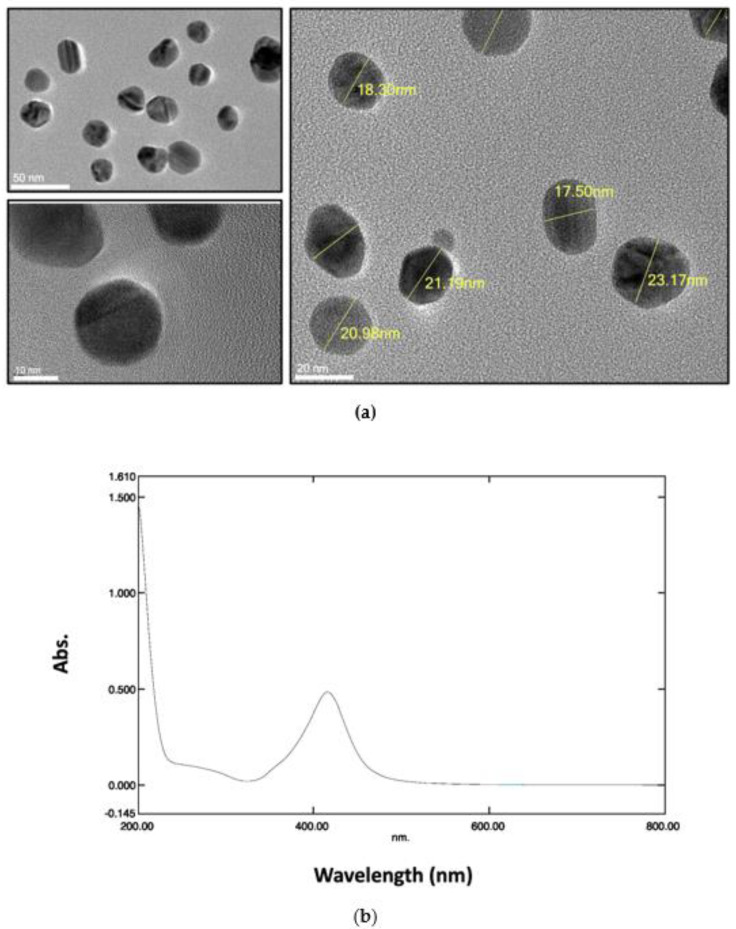
Characterization of AgNPs by transmission electron microscopy (TEM) and spectrophotometry. (**a**) Representative TEM images at different magnifications. (**b**) UV–visible spectrum of the AgNPs.

Since AgNPs were commercially available, their size and shape were assessed as a confirmatory measurement. Figure 1a shows representative transmission electron microscopy (TEM) images of AgNPs, which confirmed the spherical shape of those NPs with an average size of 15–25 nm. Figure 1b demonstrates the UV-visible spectrum of AgNPs, with a peak absorption of ~425 nm confirming the size range of the AgNPs.

Table 1 shows the hydrodynamic size, zeta potential (surface charge), and polydispersity index (PDI) for AgNPs dissolved in deionized water, cation-adjusted Mueller–Hinton broth (CAMHB), and Dulbecco’s Modified Eagle Medium (DMEM). The mean average hydrodynamic size of the AgNPs was measured as 54 ± 1 nm, 48 ± 3 nm, and 153 ± 16 nm in deionized water, CAMHB, and DMEM, respectively. The zeta potential measurements were −2.7 ± 0.9 mV, 1.7 ± 0.3 mV, and 1.9 ± 0.1 mV in deionized water, CAMHB, and DMEM, respectively (Table 1). The stability of AgNPs in different biological media represents a key parameter to maintain their activity. Indeed, previous evidence has shown that AgNPs could aggregate and/or agglomerate once they come in contact with biological media [42,43,44]. Several factors such as ionic strength, pH, surface charge, and surface coating have been shown to influence AgNPs’ stability [43,44]. Although we did not measure hydrodynamic size or surface charge over different time points, our data suggest that AgNPs tend to slightly aggregate once they are in biological media, as evidenced by the change in AgNP hydrodynamic size and/or surface charge compared to water, which was typically seen in the majority of previous nanotoxicological studies (Table 1). The PDI was less than or equal to 0.3, which indicates the homogeneity of the sample.

### 2.2. Minimum Inhibitory Concentration of AgNPs and Other Antimicrobial agents

The antimicrobial effect of AgNPs and other antimicrobials (ampicillin, kanamycin, colistin, ciprofloxacin, rifampicin, and vancomycin) was determined using the broth microdilution method against various wild-type bacterial strains, including 4 Gram-negative and 4 Gram-positive as well as 3 AMR clinical isolates, according to the M07-A10 Clinical and Laboratory Standards Institute (CLSI) protocol [45]. The results demonstrated differential susceptibilities of the wild-type bacterial strains following exposure to AgNPs. Specifically, the AgNPs were associated with MIC values ranging from 16 to 128 μg/mL against Gram-negative bacteria. *P. aeruginosa* was the most sensitive to AgNPs, while *K. pneumoniae* showed a more resistant profile (Table 2). In contrast, all Gram-positive bacterial strains exhibited consistent results with AgNPs (MIC values of ~256 μg/mL) (Table 2). Therefore, Gram-positive bacteria were resistant to AgNPs, while Gram-negative bacteria showed a less resistant pattern. The other antimicrobial agents’ MIC values were within one to two dilutions of the reference values, which is considered acceptable according to the CLSI and the European Committee on Antimicrobial Susceptibility Testing (EUCAST) (Table 2) [46,47].

The MIC values for AgNPs and other antimicrobials against the AMR *K. pneumoniae* clinical isolates are presented in Table 3. Our MIC values were compared against each antimicrobial’s CLSI breakpoint of Enterobacteriaceae species to determine the susceptibility of the bacteria to the antimicrobials. However, the CLSI established breakpoints for all used antimicrobials except vancomycin and rifampicin [47]. The MIC values were interpreted as sensitive if the MIC value was lower than the antimicrobial’s CLSI breakpoint and resistant if it was equal to or greater than the antimicrobial’s CLSI. Our results showed that all AMR *K. pneumoniae* strains were sensitive to kanamycin but resistant to ciprofloxacin (Table 3). Strains I and II were resistant to ampicillin, whereas strain III was sensitive with an MIC value of 0.5 μg/mL (Table 3). Finally, strains II and III were resistant to colistin, while strain I was sensitive (Table 3).

This study investigated the antimicrobial response of AgNPs against Gram-positive and -negative wild-type strains and showed that exposure to AgNPs resulted in significant antimicrobial activity against Gram-negative wild-type bacteria, including *E. coli*, *A. baumannii*, *K. pneumoniae*, and *P. aeruginosa*, but was associated with minimal antimicrobial activity or almost resistance to Gram-positive wild-type bacterial strains, including *S. saprophyticus*, *S. aureus*, *S. sciuri*, and *S. epidermidis* (Table 2). Therefore, only Gram-negative AMR clinical isolates (*K. pneumoniae*) were chosen for further testing (Table 3). This antimicrobial activity is consistent with previous studies that demonstrated a significant antimicrobial activity of AgNPs against Gram-negative bacteria, including against AMR *K. pneumoniae* [48,49,50,51,52]. Such an observation could be attributed to the larger negative charge or unique proteins in Gram-negative bacterial cell walls, resulting in direct interaction with AgNPs and release of silver ions (Ag^+^) [49,53]. The MIC values of AgNPs in this study were in the range of 16–128 μg/mL against Gram-negative bacteria, which is a similar range to previously reported data (Table 2) [54]. The observed variation in MIC values could be due to the inherent tolerance of the tested strains [55,56]. Importantly, the data showed that both the wild-type strains and clinical isolates of AMR *K. pneumoniae* strains had similar MIC values following their exposure to AgNPs. This was also shown previously against different AMR bacterial strains, indicating the susceptibility of the AMR bacteria to AgNPs [57,58]. Several antibacterial activities were reported for AgNPs, including direct interaction and damage of cell walls, generation of ROS, and internalization and release of Ag^+^ [53,57,58,59].

On the other hand, the results have demonstrated no difference in the MIC values (256 μg/mL) of AgNPs against all tested Gram-positive bacterial strains, including *S. aureus*, *S. saprophyticus*, *S. sciuri*, *and S. epidermidis* (Table 2). This finding is inconsistent with previous studies which found that AgNPs were effective against Gram-positive bacterial strains, with low MIC values [60,61,62]. For instance, one study reported AgNPs (5–15 nm) with MIC values of 60 and 70 μg/mL against *S. aureus* and *S. epidermidis*, respectively [60]. Another study demonstrated AgNP size-dependent MIC values against *S. aureus* ranging from 70 to 200 μg/mL [61]. However, other physicochemical properties, such as AgNPs’ surface charge and coating, could also augment the antimicrobial toxicity [62]. For example, it was reported that stabilizing AgNPs by coating the nanoparticle surface with carboxymethyl cellulose (CMC), a protective agent used to control and maintain particle size (i.e., minimize aggregation and agglomeration) and dispersion stability, enhanced their antimicrobial activity [60]. Additionally, the electrostatic attraction between negatively charged microorganism membranes and positively charged Ag^+^ might play an essential role in determining the strength of the antimicrobial activity of AgNPs. Altogether, the findings of this study are consistent with previous reports and demonstrate the favorability of AgNPs’ antibacterial activity against Gram-negative bacterial strains [49,53,62,63].

### 2.3. Synergy between AgNPs and Tested Antimicrobials

Finding synergistic combinations between different antimicrobials and AgNPs is key for tackling AMR bacteria. Therefore, the potential synergy between AgNPs and different antimicrobials was investigated against a wild-type (*E. coli*) strain and three AMR clinical isolates of *K. pneumoniae* using the checkerboard method and the fractional inhibitory concentration (FIC) index, as mentioned earlier. The FIC index is a mathematical expression used to evaluate the impact of drug combinations and determine whether synergy exists. For this study, the ΣFIC result was interpreted as synergism if the values were ≤0.5, indifference if values > 0.5 and ≤4, and antagonism if values > 4. The lowest ΣFIC value of each combination was considered a representative result for that combination [64]. Table 4 shows the MIC values for each antimicrobial alone (MIC of AM alone), each antimicrobial in combination (MIC of AM in combination), AgNPs alone (MIC of AgNPs alone), and AgNPs in combination (MIC of AgNPs in combination), as well as ΣFIC values for wild-type *E. coli* and AMR strains. Combinations of AgNPs with kanamycin or colistin against wild-type *E. coli* resulted in synergy, with remarkable ΣFIC values equal to 0.1 (Table 4). The combination of AgNPs and rifampicin displayed a synergistic effect in which rifampicin remarkably reduced the MIC value of AgNPs from 128 to 16 μg/mL (Table 4). Lastly, the combination of AgNPs and vancomycin also produced a synergistic effect with a ΣFIC value of 0.5, while combinations of AgNPs and ampicillin or ciprofloxacin resulted in no difference, with ΣFIC values equal to 1 and ≤0.7, respectively (Table 4).

The findings showing significant positive synergistic interactions between AgNPs in combination with kanamycin, colistin, rifampicin, or vancomycin encouraged us to examine whether these combinations can also be synergistic against more challenging clinical isolates of Gram-negative bacteria. Therefore, we tested these combinations against three clinical isolates of *K. pneumoniae.* For strain I, combinations of AgNPs and kanamycin, colistin, ciprofloxacin, or rifampicin demonstrated synergistic activities with ΣFIC values between 0.3 and 0.5 (Table 4). In contrast, treatment with AgNPs and ampicillin or vancomycin displayed indifferent activities with ΣFIC values of 0.7 and 0.6, respectively (Table 4). For strain II, combinations of AgNPs and kanamycin or colistin exhibited a synergistic effect, with ΣFIC values of 0.1 and 0.3, respectively. Furthermore, treatment with AgNPs and colistin resulted in a reduction in AgNPs’ MIC value from 64 to 4 μg/mL (Table 4). The combination of AgNPs and rifampicin resulted in synergistic activity as well. with a ΣFIC value of 0.5 (Table 4). Treatment with AgNPs and vancomycin also demonstrated synergy and reduced the MIC values of AgNPs and vancomycin from 64 to 32 μg/mL and from 1024 to 64 μg/mL, respectively (Table 4). On the other hand, treatment with AgNPs and ampicillin or ciprofloxacin resulted in ΣFIC values of > 0.5 and < 4, respectively, which indicate indifferent activity (Table 4). Finally, for strain III, the data demonstrated a similar pattern to that for strain II—that is, synergy was found among the combinations of AgNPs and kanamycin, colistin, rifampicin, or vancomycin, whereas treatment with AgNPs and ampicillin or ciprofloxacin showed no difference (Table 4). The synergy between AgNPs and kanamycin (ΣFIC value of 0.3) resulted in a reduction in the AgNP and kanamycin MIC values from 128 to 16 μg/mL and from 4 to 1 μg/mL, respectively (Table 4). Treatment with AgNPs and colistin or rifampicin demonstrated a synergistic effect with ΣFIC values of 0.5 and 0.3, respectively (Table 4). The combination of AgNPs and vancomycin resulted in a reduction in MIC values from 128 to 8 μg/mL for AgNPs (ΣFIC = 0.1). It is worth noting that there were no antagonistic effects among the three AMR strains with all tested treatments.

The findings of this study demonstrated synergy between the combinations of AgNPs and four different antimicrobials from different pharmacological classes, including kanamycin, colistin, rifampicin, and vancomycin, against wild-type *E. coli* and the three AMR clinical isolates of ESBL-positive *K. pneumoniae* strains (except for vancomycin in strain I) (Table 4). The results suggest susceptibility differences among AMR isolates in response to the treatment with AgNPs, antimicrobials, or combinations of AgNPs and antimicrobials, which could be attributed to varying resistance genes’ makeup. However, and most importantly, these AMR strains all remain susceptible to combinations of AgNPs and kanamycin, colistin, rifampicin, or vancomycin (except for strain I and vancomycin), indicating that the AgNPs might work through mechanism(s) that remain susceptible in AMR bacteria.

To the best of our knowledge, this is the first study that evaluates the potential synergy of combinations between AgNPs and different conventional antimicrobials against clinical isolates of AMR *K. pneumoniae*. However, the findings reported in this study are consistent with the previous literature with regard to the development of synergy between AgNPs and the other antimicrobials against a broad range of bacterial strains. One key combination that produced consistent synergy against all AMR strains was AgNPs and kanamycin. Indeed, previous reports have demonstrated the effectiveness of this combination against a variety of bacterial strains [60,65,66]. For instance, one study showed that combining AgNPs and kanamycin could overcome kanamycin resistance against *P. aeruginosa* [66]. Another study demonstrated that combining AgNPs with kanamycin tends to inhibit the growth of *E. coli*, *Salmonella enterica Serovar Typhimurium*, and *S. aureus* at a percentage of ~95% compared to ~30% with kanamycin monotherapy [67]. A second combination that produced synergy against both wild-type and AMR strains was AgNPs and colistin. Previous studies demonstrated excellent synergistic activity between colistin and AgNPs against different bacterial strains, such as *E. coli, P. aeruginosa*, and AMR *A. baumannii,* with MIC values two- to fourfold lower than that of colistin alone [68,69]. A third combination that produced synergy in both wild-type *E. coli* and AMR *K. pneumoniae* bacterial strains was AgNPs and rifampicin. The co-treatment with AgNPs increased the susceptibility of the bacteria to rifampicin, with a significant reduction in MIC values (from 8 to 1 µg/mL in the wild-type strain, from 16 to 4 µg/mL in strains I and II, and from 0.0005 to 0.002 µg/mL in strain III) (Table 4). Such synergy was reported in several studies against several bacterial strains [38,65,70,71,72,73]. For instance, one study found that combining AgNPs (at 25 μg/disc) and rifampicin (5 μg/disc) resulted in potent antimicrobial activity against multiple pathogens, including *B. cereus, L. monocytogenes, S. aureus, E. coli, and S. Typhimurium.* Neither AgNPs nor rifampicin alone have demonstrated such antimicrobial activity at the same doses [65]. Lastly, the fourth combination of AgNPs and vancomycin produced synergy in three strains, including the wild-type and two AMR strains (Table 4). Vancomycin alone showed very low antibacterial activity against *E. coli*, as with most of Gram-negative bacteria, due to the intrinsic resistance of bacteria against this medication [74]. However, it is evident from the current study that the combination with AgNPs improved its antibacterial activity (Table 4), which is in agreement with the previous literature [68,75,76,77]. One previous study reported that vancomycin was able to produce an antimicrobial response against *E. coli* only when it was used in combination with AgNPs [77]. Altogether, the findings in this study demonstrate the key role of using AgNPs in combination with conventional antimicrobials as evidenced by the improved efficacy of different classes of antimicrobials against AMR bacterial strains.

As mentioned previously, the antimicrobial properties of AgNPs can be attributed to multiple different mechanisms, including disruption of the bacterial cell wall, generation of ROS, and internalization and release of Ag^+^ (Trojan horse mechanism) [27,28,29,30]. Therefore, their synergistic activity with different antimicrobials (which possess different antibacterial activities) could be due to one or more mechanisms. Gram-negative bacteria are characterized by the presence of an outer membrane composed of lipopolysaccharide (LPS), which functions as a protective layer that limits the penetration of hydrophobic antimicrobials and those with large molecular weights [78]. Both kanamycin and rifampicin are hydrophobic antimicrobials, while vancomycin has a large molecular weight (1,449.3 gm/mol) [79,80]. One key mechanism through which AgNPs could improve the efficacy of kanamycin (protein synthesis inhibitor), rifampicin (RNA synthesis inhibitor), and vancomycin (cell wall synthase inhibitor) against Gram-negative bacteria is direct interaction with and disruption of bacterial outer cell membranes (i.e., increase in membrane permeability). This might facilitate the entry of antimicrobials into bacteria, hence acting on their intracellular targets [51,67,81]. In the case of cell membrane disruptor antimicrobials, such as colistin, the synergy with AgNPs may be attributed to the combined disruption of bacterial cell membranes. Although the molecular mechanisms underlying the synergistic activity of the tested combinations were not investigated in this study, it is worth noting that other studies have investigated the specific mechanisms of AgNP-mediated suppression of antimicrobial resistance. For instance, it was shown that AgNPs could suppress antimicrobial resistance through activation of efflux pump systems (reported in *Enterobacteriaceae*) against several antimicrobials, including kanamycin, colistin, and rifampicin [82,83,84]. Another recent study investigating the underlying antimicrobial mechanism of lysozyme-coated AgNPs against AMR *K. pneumoniae* (MGH78578, ATCC^®^ 700721) using transcriptomics analysis identified oxidative stress and a triclosan-like antibacterial mechanism [52]. Altogether, emerging research has started to unravel the underlying molecular mechanisms of AgNP antimicrobial properties. However, further investigations and understanding of the underlying molecular mechanisms of our synergistic combinations are warranted.

Finally, the data in this study showed that the combinations of AgNPs and ampicillin (peptidoglycan cross-linking inhibitor) or ciprofloxacin (DNA gyrase inhibitor) produced no synergy against the tested bacterial strains (wild-type and AMR isolates). Previous studies demonstrated inconsistent findings for AgNP-based combinations with those two antimicrobials. One study showed that AgNP-based combinations rendered several pathogens susceptible to ampicillin and ciprofloxacin, including *Streptococcus mutans*, *Streptococcus oralis*, and *Aggregatibacter actinomycetemcomitans* [85]. Other studies reported synergy only between AgNPs and ampicillin against free-living *P. aeruginosa*, but not ciprofloxacin, as well as synergy in a AgNP-based combination with ampicillin against only one bacterial strain (i.e., *A. baumannii*) among twelve other tested strains [73,86]. These inconsistencies could be due to variations in the physicochemical properties of the AgNPs used in those studies. For instance, it was previously reported that coating AgNPs with polyvinylpyrrolidone (PVP) could impact the antimicrobial activity of AgNPs [39,87].

### 2.4. Cytotoxicity of AgNP-Based Combinations with Synergy in Mammalian Cells 

**Figure 2:**
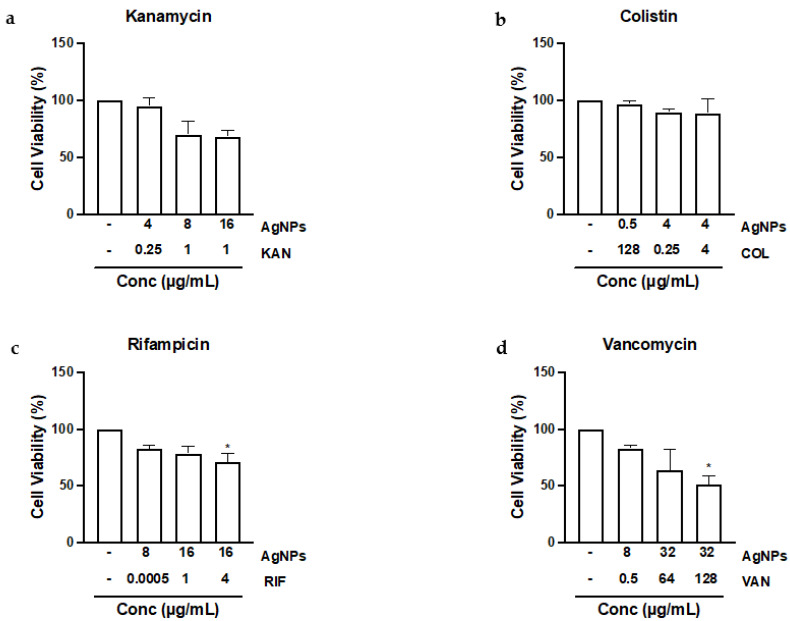
Cytotoxicity of tested AgNP-based combinations with synergy. HepG2 cells were exposed to AgNPs in the presence of the antimicrobials (**a**) kanamycin, (**b**) colistin, (**c**) rifampicin, and (**d**) vancomycin for 24 h at the same concentrations that produced synergy, and viability was assessed using the MTT assay based on the formation of formazan salts. Significant changes are marked with an asterisk (*), *p*-value < 0.05, using a one-way ANOVA with the Bonferroni post hoc test (*n* ≥ 3).

One critical aspect of newly developed therapeutic agents or materials is their human safety. Indeed, ensuring the safety of ENMs represents one of the major challenges in the field of nanotoxicology [21,88]. AgNPs, like many other inorganic ENMs, have been demonstrated to be associated with toxicological manifestations, particularly at high concentrations, which is largely attributed to ENM physicochemical properties such as size, shape, and surface charge [89]. Therefore, it is important to assess the potential toxicity of AgNPs in mammalian cells, especially at the concentrations used in the tested combinations. Therefore, to gain some insight on the potential toxicity of the used AgNP-based combinations, HepG2 cells were exposed to AgNPs in combination with the antimicrobials that produced synergy. HepG2 cells, a widely used human hepatocyte model, were used since the liver is the primary organ for detoxification of xenobiotics and also a key organ of the reticuloendothelial system (RES) [90,91]. The findings indicated no to minimal cellular toxicity at most of the concentrations used in the synergistic combinations (Figure 2). Furthermore, the data showed significant toxicity for AgNPs at concentrations of ≥16 µg/mL (Figure S1). It is worth noting that numerous previous studies investigating AgNP antimicrobial properties lacked a concurrent cytotoxicity assessment, therefore making comparison between studies almost impossible [35,67,73,92]. Altogether, the cytotoxicity study suggests that exposure to the AgNPs at concentrations of <16 µg/mL, which represent the majority of the synergistic combinations used in this study, are overall tolerable. Nevertheless, carrying out an in vivo toxicity study is necessary to make a conclusive statement regarding the safety of the used AgNP-based combinations.

## 3. Materials and Methods

### 3.1. Materials

AgNPs (15 nm, Cat No. US7091) were purchased from US Research Nanomaterials, Inc. (Houston, TX, USA). Cation-adjusted Mueller–Hinton broth (CAMHB) (Sentmenat, Barcelona, Spain) and Dulbecco’s Modified Eagle Medium (DMEM) (Corning Inc., Corning, NY, USA) were used in the microbiology and cell culture studies, respectively. All antimicrobial agents used in this study were purchased from the Toronto Research Chemicals Company (North York, YTO, Canada).

### 3.2. Characterization of AgNPs

The hydrodynamic size (nm), zeta potential (mV) (surface charge), and polydispersity index (PDI) of AgNPs were measured using a Zetasizer system (Malvern, Westborough, MA) in deionized water, CAMHB, or DMEM. A transmission electron microscope (TEM, JEOL JSM-1010, Akishima, Tokyo, Japan) was used to confirm the size and the shape of the AgNPs.

### 3.3. Bacterial Strains

The laboratory non-pathogenic bacterial strains used for the in vitro microbiological studies were obtained from the American Type Culture Collection (ATCC) (Manassas, VA, USA), which included the following bacteria: *E. coli* (ATCC 25922), *Acinetobacter baumannii* (*A. baumannii,* ATCC BAA-747), *K. pneumoniae* (ATCC 13883), *P. aeruginosa* (ATCC 27853), *Staphylococcus saprophyticus* (*S. saprophyticus,* ATCC 49453), *Staphylococcus aureus* (*S. aureus,* ATCC 29213), *Staphylococcus sciuri* (*S. sciuri,* ATCC 29061), and *Staphylococcus epidermidis* (*S. epidermidis,* ATCC 12228). Three AMR *K. pneumoniae* clinical isolates were selected for checkerboard experiments based on their resistant phenotypes. These strains were isolated and collected from King Khalid University Hospital (KKUH), Riyadh, Saudi Arabia (Table 5).

### 3.4. Minimum Inhibitory Concentration (MIC) of AgNPs and Other Antimicrobial agents

The MIC can be defined as the lowest concentration of an antimicrobial agent that is required to inhibit the visible growth of a bacterium [7]. The MIC values for AgNPs and other antimicrobial agents against the laboratory bacteria and AMR clinical isolates were determined by using the broth microdilution method based on the M07-A10 CLSI protocol [93]. Briefly, 50 µL of CAMHB was added to all wells of a sterile 96-well plate except column No. 12, where 100 µL of CAMHB was added. Next, AgNPs (5 mg/mL) or other antimicrobials were added to column No. 12 and diluted 2-fold into the proceeding columns until column No. 2 to give a range of different concentrations of the antimicrobials. Working stocks of AgNPs (i.e., serial dilutions in sterile deionized water) were prepared from original stock (5 mg/mL in water) with careful and thorough mixing (i.e., vortexing and pipetting) while preparing serial dilutions and after adding AgNPs to CAMHB. Column No. 1 lacked any antimicrobial and was used as a bacterial growth control. After that, 50 µL of inoculum of the bacterial culture was added to each well of the 96-well plate. The final concentrations of AgNPs or other antimicrobials ranged from 0.25 to 128 μg/mL from column No. 12 until column No. 2, respectively. Finally, the plate was incubated with agitation (220 rpm) at 37 °C for 18 h. The MIC was determined by finding the lowest concentration that inhibited the visible bacterial growth in each row for each tested antimicrobial agent. A spectrophotometer (600 nm) was used to confirm bacterial growth inhibition.

All ATCC bacteria and AMR *K. pneumoniae* clinical isolates were prepared by inoculating colonies in 0.5 McFarland of CAMHB to produce 10^8^ colony-forming units/mL (CFU/mL), followed by culture on blood agar and incubation overnight at 37 °C.

### 3.5. Synergistic Effect of AgNP-Based Combination with Other Antimicrobial agents

The presence of potential synergistic activities between AgNPs combined with other antimicrobial agents was evaluated by the checkerboard method against wild-type and selected AMR clinical isolates. The determination of the tested concentrations in the checkerboard depended on the MIC values. Two concentrations above the MIC value were chosen to account for the antagonistic effects, and the other chosen concentrations accounted for the synergistic and indifference effects. The general approach of the checkerboard assay resembles the microdilution method used to determine the MIC value. However, the two antimicrobials were diluted in two distinct directions to make the concentrations intersect with each other. Hence, AgNPs were diluted one way (i.e., from columns 11 to 4), while the other antimicrobial was diluted the other way (i.e., from row H to row B), and then, the bacterial inoculum was added similarly to the MIC plate. Wells with untreated bacteria or medium were considered as positive and negative controls, respectively. Finally, the concentrations of the two agents that showed complete bacterial growth inhibition were recorded.

Interpretation of the experimental results was conducted using the fractional inhibitory concentration (*FIC*) index, a mathematical expression used to evaluate the impact of drugs’ combination and determine whether a synergistic effect exists or not [94]. The ***Σ****FIC* index was calculated using the following formula:

(1) $$\Sigma FIC\;=\;\frac{MIC\;of\;agent\;A\;in\;combination}{MIC\;of\;agent\;A\;alone}+\frac{MIC\;of\;agent\;B\;in\;combination\;}{MIC\;of\;agent\;B\;alone}$$

The *ΣFIC* result was interpreted as synergism if the values were ≤0.5, indifference if values were >0.5 and ≤4, and antagonism if values were >4.

### 3.6. Cytotoxicity Evaluation of AgNP-Based Synergistic Combination with Other Antimicrobial Agents

HepG2 (ATCC HB-8065), a human liver cell model, was used to evaluate the safety of the AgNPs with/without the antimicrobials. The HepG2 cell line was purchased from ATCC (Manassas, VA, USA). The HepG2 cells were cultured and propagated according to the ATCC guidelines [95]. In short, the cells were cultured at 37 °C and 5% CO_2_ in DMEM supplemented with 10% fetal bovine serum (FBS) and 100 U penicillin/100 μg streptomycin/mL (Corning Inc., Corning, NY, USA). Cell viability was determined by the MTT assay based on the cell mitochondrial activity. Cells were cultured in 96-well plates in serum-supplemented medium to ~80% confluency. Cells were then treated with AgNPs at final concentrations of 0.5–32 µg/mL in the presence or absence of antimicrobial combinations for 24 h in serum-free media. Working stocks of AgNPs were prepared from original stock (5 mg/mL) in sterile deionized water with careful and thorough mixing (i.e., vortexing and pipetting). MTT (500 μg/mL) was added to the cells, which were incubated at 37 °C and 5% CO_2_ until color development and crystal formation. Formazan crystals were then dissolved using isopropanol, and the absorbance was measured using a BioTek Synergy^TM^ HT spectrophotometer at a wavelength of 570 nm (BioTek, Winooski, VT, USA). The cell survival rates were calculated and plotted as percentage viability using the relative absorbance equation as follows:

(2) $$\mathrm{Relative}\;\mathrm{absorbance}=\frac{Absorbance\;of\;sample}{Absorbance\;of\;control}\;\times \;100$$

### 3.7. Statistical Analysis

All experiments were performed at least in 3 independent replicates, and the results are presented as mean ± standard error (SE). Data points were analyzed by GraphPad Prism software (GraphPad Inc., San Diego, CA, USA). A one-way analysis of variance (ANOVA) and the Bonferroni post hoc test were used to analyze multi-treatment groups, whereas Student’s *t*-test was employed to compare two treatment groups. A *p*-value of <0.05 was considered statistically significant.

## 4. Conclusions

AMR bacteria have emerged as a global health threat, and currently, there is an overall shortage in the development of new antimicrobials. In recent years, AgNPs have shown promising antimicrobial properties; however, the relevant dosage of AgNPs in combination with different antimicrobials is rarely known. Additionally, the cytotoxicity of AgNPs within synergistic combinations to mammalian cells is seldom reported. Therefore, this study sought to investigate AgNP-based combinations with different classes of antimicrobials against wild-type Gram-positive and -negative bacterial strains as well as clinically isolated AMR *K. pneumoniae* bacteria. This study concluded that four out of the six tested AgNP-based combinations (i.e., kanamycin, colistin, rifampicin, and vancomycin) had synergistic activities against all tested Gram-negative bacteria (except vancomycin in AMR strain I). One important finding is that the MIC values of both wild-type and AMR bacteria were similar following exposure to AgNPs, hence suggesting that AgNPs act via a mechanism or multiple mechanisms through which the tested bacteria remain susceptible to them. However, further studies are required to confirm this hypothesis. Finally, exposing the AgNP-based combinations at the synergistic concentrations to mammalian cells, namely HepG2, demonstrated minimal toxicity, particularly at concentrations < 16 µg/mL.

Although the previous reports discussed earlier demonstrated great antimicrobial potential for AgNPs against different types of bacteria, including AMR bacteria, there remain inconsistencies with regard to the used strains, the physicochemical properties of AgNPs, the dosing and antimicrobial combinations, and the cytotoxicity in mammalian cells [35,36,37,38,39]. The novelty of this work is in its investigation of the potential synergy of commercially available AgNPs in combination with different classes of antimicrobials for the treatment of locally isolated AMR *K. pneumoniae*, since emerging evidence is demonstrating the increasing prevalence of local infections caused by AMR *K. pneumoniae* [15]. Furthermore, in vitro and in vivo toxicity assessments have often been lacking in previous studies [35,67,73,92]. Therefore, this study attempted to address such inconsistencies by investigating the potential use of AgNPs in combination with representative antimicrobials from different classes against a wide range of standard bacterial strains and locally isolated AMR bacteria that represent a challenge to clinicians. In addition, this study evaluated the cytotoxicity of the synergistic combinations to provide some insight into the translational potential of the findings reported in this study. Future studies using relevant animal models are warranted to confirm and consolidate the findings reported in this study.

## Figures and Tables

**Table 1:** AgNP characterization in water, CAMHB, and DMEM. Results are shown as mean ± SE.

ENMs	Medium	Hydrodynamic size (nm)	Zeta potential (mV)	PDI
AgNPs	Water	54 ± 1	−2.7 ± 0.9	0.22 ± 0.01
	DMEM	153 ± 16	1.9 ± 0.1	0.34 ± 0.02
	CAMHB	48 ± 3	1.7 ± 0.3	0.29 ± 0.01

**Table 2:** MIC values of AgNPs and antimicrobial agents against Gram-negative and -positive wild-type bacteria.

Gram-Negative Bacteria							
Organism	MIC μg/mL						
	AgNPs	AMP	KAN	VAN	CIP	COL	RIF
*E. coli* (ATCC 25922)	64	2	2	256	0.015	2	4
*K. pneumoniae* (ATCC 13883)	128	>256	1	>256	0.25	ND	16
*P. aeruginosa* (ATCC 27853)	16	>256	>256	>256	>0.25	0.5	16
*A. baumannii* (ATCC BAA-747)	32	16	4	>256	0.25	1	0.5
**Gram-positive bacteria**							
*S. aureus* (ATCC 29213)	256	0.5	4	1	>0.25	>256	>0.25
*S. saprophyticus* (ATCC 49453)	256	1	0.5	1	0.25	64	>0.25
*S. sciuri* (ATCC 29061)	256	2	8	0.5	0.25	ND	<0.25
*S. epidermidis* (ATCC 12228)	256	ND	ND	2	0.25	>256	<0.25

Ampicillin, AMP; kanamycin, KAN; colistin, COL; ciprofloxacin, CIP; rifampicin, RIF; vancomycin, VAN; not determined, ND.

**Table 3:** MIC values of AgNPs and antimicrobial agents against AMR strains.

AMR strains							
Organism	MIC μg/mL						
	AgNPs	AMP	KAN	VAN	CIP	COL	RIF
*K. pneumoniae* Strain I	64	4096	4	1024	4	1	16
*K. pneumoniae* (ESBL) Strain II	64	4096	4	2048	1	64	16
*K. pneumoniae* (ESBL) Strain III	128	0.5	4	8	16	256	0.002
**CLSI breakpoints (μg/mL)**	**NR**	**≥32**	**≥64**	**NR**	**≥1**	**≥4**	**NR**

Ampicillin, AMP; kanamycin, KAN; colistin, COL; ciprofloxacin, CIP; rifampicin, RIF; vancomycin, VAN; not determined, ND; extended spectrum β-lactamase (ESBL). The bold text indicates antimicrobial resistance.

**Table 4:** The checkerboard results for AgNP-based combinations with different antimicrobials against a wild-type (*E. coli*) strain and AMR *K. pneumoniae* strains.

Combination	MIC of AgNPs Alone	MIC of AgNPs in Combination	MIC of AM Alone	MIC of AM in Combination	ΣFIC	Effect
**Wild-type (*E. coli*) strain**						
AMP + AgNPs	64	32	4	2	1	Indifference
KAN + AgNPs	64	4	2	0.25	0.1	**Synergism**
COL + AgNPs	128	4	2	0.25	0.1	**Synergism**
CIP + AgNPs	128	32	0.016	0.008	0.7	Indifference
RIF + AgNPs	128	16	8	1	0.2	**Synergism**
VAN + AgNPs	128	32	512	128	0.5	**Synergism**
***K. pneumoniae*** **Strain I**						
AMP + AgNPs	64	16	4096	2048	0.7	Indifference
KAN + AgNPs	64	8	4	1	0.3	**Synergism**
COL + AgNPs	32	8	1	0.25	0.5	**Synergism**
CIP + AgNPs	128	64	4	0.065	0.5	**Synergism**
RIF + AgNPs	64	16	16	4	0.5	**Synergism**
VAN + AgNPs	64	8	1024	512	0.6	Indifference
***K. pneumoniae*** **(ESBL) Strain II**						
AMP + AgNPs	64	16	4096	2048	0.7	Indifference
KAN + AgNPs	64	8	4	1	0.3	**Synergism**
COL + AgNPs	64	4	64	4	0.1	**Synergism**
CIP + AgNPs	64	32	4	1	0.75	Indifference
RIF + AgNPs	64	16	16	4	0.5	**Synergism**
VAN + AgNPs	64	32	1024	64	0.5	**Synergism**
***K. pneumoniae*** **(ESBL) Strain III**						
AMP + AgNPs	128	64	0.5	0.125	0.75	Indifference
KAN + AgNPs	128	16	4	1	0.3	**Synergism**
COL + AgNPs	128	0.5	256	128	0.5	**Synergism**
CIP + AgNPs	128	16	32	16	0.625	Indifference
RIF + AgNPs	128	8	0.002	0.0005	0.3	**Synergism**
VAN + AgNPs	128	8	8	0.5	0.1	**Synergism**

Ampicillin, AMP; kanamycin, KAN; colistin, COL; ciprofloxacin, CIP; rifampicin, RIF; vancomycin, VAN; antimicrobial, AM. Synergism ≤ 0.5; indifference > 0.5 and ≤4; antagonism > 4.

**Table 5:** AMR *K. pneumoniae* strains used in this study.

Organism	Resistant Phenotypes
*K. pneumoniae* Strain I	Ampicillin, cephalosporin, ciprofloxacin, vancomycin
*K. pneumoniae* (ESBL) Strain II	Ampicillin, cephalosporin, ciprofloxacin, colistin, vancomycin
*K. pneumoniae* (ESBL) Strain III	Cephalosporin, ciprofloxacin, ertapenem, tobramycin, Trimethoprim/ Sulfamethoxazole, colistin, vancomycin

Extended spectrum β-lactamase (ESBL).

## Data Availability

Not applicable.

## Supplementary Materials

The following supporting information can be downloaded at: https://www.mdpi.com/article/10.3390/antibiotics11091219/s1, Figure S1. Cytotoxicity following exposure to silver nanoparticles. HepG2 cells were exposed to AgNPs for 24 h at a range of concentrations to assess cytotoxicity following exposure to AgNPs alone. Cell viability was assessed using the MTT assay based on the formation of formazan salts. Significant change from the non-treated control is marked with an asterisk (*), *p*-value < 0.05, using a one-way ANOVA with the Bonferroni post hoc test (n ≥ 3).
