# Supplementary material for: Silver Nanoparticle-Based Combinations with Antimicrobial Agents against Antimicrobial-Resistant Clinical Isolates
Source: Antibiotics (Basel). 2022 Sep 8;11(9):1219. doi: 10.3390/antibiotics11091219 (PMC9495250; doi:10.3390/antibiotics11091219)
Supplement: Supplementary file 1 [file antibiotics-11-01219-s001.zip › antibiotics-1876320-supplementary.pdf]

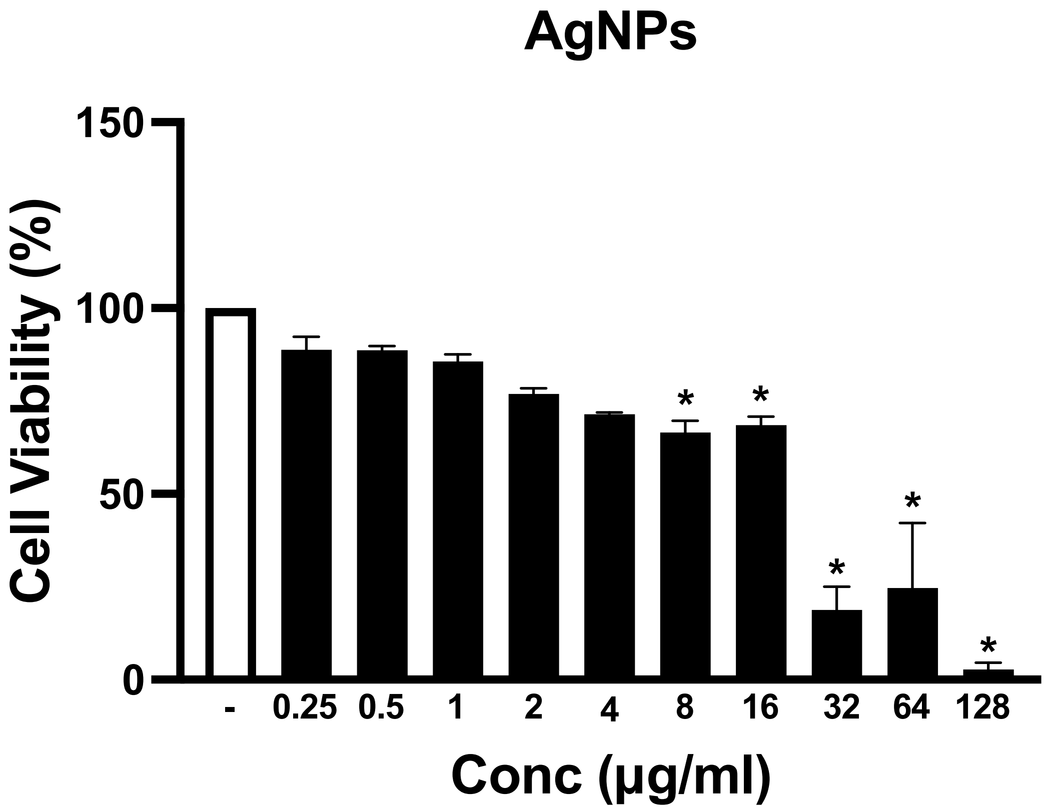

Figure S1. Cytotoxicity following exposure to silver nanoparticles. HepG2 cells were exposed to AgNPs for 24 h at a range of concentrations to assess cytotoxicity following to exposure to AgNPs alone. Cell viability was assessed using the MTT assay based on the formation of formazan salts. Significant change from nontreated control is marked with an asterisk (*) *p*-value < 0.05, using a one-way ANOVA with Bonferroni post-hoc test (n ≥ 3).
